# Two cities, two stages in transforming society—a mixed methods study comparing doctors’ adoption of mobile apps for communication with patients in Hangzhou and Yancheng, China

**DOI:** 10.3389/fpubh.2024.1320949

**Published:** 2024-02-05

**Authors:** Dongjin Chen, Zhenhua Su, Zheng Gu

**Affiliations:** ^1^Institute for Social Governance and Communication Innovation of Zhejiang, Communication University of Zhejiang, Hangzhou, China; ^2^College of Media and International Culture, Zhejiang University, Hangzhou, China; ^3^School of Public Affairs, Zhejiang University, Hangzhou, China

**Keywords:** medical platform apps, doctor-patient communication, transforming society, China, mixed-methods

## Abstract

**Objectives:**

Mobile apps have become commonplace in doctor-patient communication over the last 20 years. Doctors mainly use two kinds of app, social networking apps (i.e., WeChat) and medical platform apps (i.e., Haodf). The purpose of this study was to investigate whether the attributes of social interaction in local society impact doctors’ choice of mobile apps to communicate with patients. This article addresses two research questions: (a) To what degree do doctors’ adoption patterns in different societies differ? (b) Why do doctors choose certain mobile apps to communicate with patients?

**Methods:**

This study employed a mixed methods research design to analyze doctor’s adoption behavior patterns in two cities, Hangzhou (HZ) and Yancheng (YC), which represent two stages in transforming society. Various patterns, measured as the percentage of doctors who utilize the medical platform app of Haodf among all doctors and the average service counts per doctor, were compared in three groups of tertiary hospitals: the top ones in HZ, the average ones in HZ, and the average ones in YC. We also conducted thematic content analysis of qualitative data from semi-structured interviews with 20 purposely selected doctors in the two cities.

**Results:**

The percentages of doctors who have adopted the app of Haodf from the three groups of tertiary hospitals were 49.97%, 41.00%, and 32.03%, with an average service counts per doctor of 261, 182 and 39, respectively. According to the interviewees, doctors from YC are more likely to use social networking apps to communicate with patients than their HZ counterparts to maintain social connections with their relatives, friends, colleagues, and others.

**Conclusion:**

This study demonstrates that doctors’ choices of mobile apps are dependent upon social context. In traditional society, where people have close ties, the logic of using social networking apps lies in doctors’ need to maximize the utility of their knowledge by maintaining social connections with others. In modern society, where the close ties between people have gradually weakened, the logic of using medical platform apps lies in doctors’ needs for reputation marketing, either for themselves or for institutions, their affiliated departments or hospitals.

## 1 Introduction

High quality doctor-patient communication is essential for promoting patient health. The introduction of mobile apps, such as online medical platforms, social networking, and short video apps, have broken geographical boundaries and provided conveniences to both parties (1, 2). Among these choices, social networking apps (i.e., WeChat) and the online medical platform apps (i.e., Haodf) are two main choices.

Most existing studies on doctors’ adoption behavior assume that they might use only one kind of app to communicate with patients and focus on doctors’ adoption behavior of medical platform apps in general or one particular app, such as the Haodf app, China’s most popular commercial medical platform app (2, 3). Few studies have explored the concurrent use of social networking apps and online medical platforms. In current studies, there are a variety of terms describing doctors’ choice of mobile app to communicate with patients, including “online health community” (4), “mHealth apps” (2), and “online medical platform” (5). There is an implicit assumption in these studies that doctors’ online communication is as professional as their offline communication with patients in hospital settings; however, Chinese doctors’ communication with patients are not as professional as that of their counterparts in other countries.

In addition to online medical platform apps, doctors in China also use personal social networking accounts or phone calls to communicate with their patients. They often sacrifice their personal off-duty time to respond to messages or calls from their patients. It has been acknowledged that this phenomenon is not only popular among general practitioners but also among specialists in tertiary hospitals (6). For Chinese doctors, the boundary between one’s professional and personal life is unclear, and professional activities are combined with social obligations.

Understanding the multiple choices of patient-doctor communication applications presents a different perspective to understanding the patient-doctor relationship in general. In China, the perception of doctors is multifaceted. In one respect they are seen as cold and indifferent, which strains their relationship with their patients (7). Conversely, in some contexts, they are widely depicted as “family members” by the media. A searching of “doctors” and “like a family member” (in Chinese) in the Google search engine yields more than 800,000 results, mostly news reporting doctors’ dedication to patients at the price of their personal time. Thus, the doctor-patient relationship is as diverse as the doctors’ choice of mobile apps to communicate with patients.

The complicated doctor-patient relationship is related to the particular social context in which it takes place. We employed the perspective of the transforming society to understand the wholistic nature of particular social contexts of cities in China. This perspective regards the transformation as a long process from traditional society to modern one. Represented by Parsons and Lerner, many sociologists studying the difference between traditional society and modern society shared a view that individuals in modern society value impartiality and independence more than those in traditional society (8, 9). In particular, Parsons argued that the dichotomy between universalism and particularism is one of several dimensions to understand the different social interaction patterns between two societies (8). In modern society, individuals are treated or judged based on the universal rules, unlike the criteria based on the potential connections attached to them in traditional society (8). Lerner found that the evidence of the transforming social attributes of local residents in a small village in Turkey (9). These views could also find evidences in China, which is also experiencing a transforming process.

Studies on Chinese society, particularly its social interactions in traditional society, emphasize that people seek social services via social connections (*guanxi*) with each other (10, 11). According to Fei Xiaotong, the exchanged benefits are shared among known ones via different kinds of connections, such as kinship or friendship, forming acquaintance society (12). This pattern of behavior is different from the counterpart in modern society (stranger society), where strangers are able to exchange their benefits based on rules and monetary incentives (12). Studies suggest that China is shifting from traditional society (acquaintance society) to modern one (stranger society). Yet, many people continue to interact with each other as if it were still traditional society (11, 13). In medical service provision, relying on social connections is not rare for patients to seek better or more trustful doctors in China (14, 15).

Researchers have used this perspective to explore the doctor-patient relationship in the offline service, finding that patients turn to social connections to seek more trustworthy medical services (16, 17). We applied this perspective to explore the possible influence of *guanxi* on the doctor-patient communications in today’s China as mobile apps are gaining more popularity. A transforming society can be seen as a spectrum with traditional society on one end and modern one on the other. To be sure, even the most modern city in China might retain aspects of traditional society, and the most traditional city will have some elements of modern society. We choose two cities, Hangzhou (HZ), a modern city, and Yancheng (YC), a traditional city, which represent the two stages in this transforming process.

This study’s purpose is to determine whether regional differences impact doctors’ adoption of mobile apps to communicate with patients by addressing two research questions: (a) To what degree do doctors’ adoption patterns in the two cities differ? (b) Why do doctors choose certain mobile apps to communicate with patients?

## 2 Materials and methods

### 2.1 Research design

We employed mixed methods, descriptive statistics from purposely selected cases and thematic content analyses of semi-structured interviews to explore the different patterns of doctors’ usage of apps in the two cities and the reasons behind them. Descriptive statistics provide summarized results of the collected data. Thematic content analyses, which have been used to conduct explorative studies, are ideal for studying doctor-patient communication (18), which is replete with privacies and personal perceptions (19). Du and colleagues also used the qualitative method to explore this complex relationship (20).

Descriptive statistics were utilized in our study to display doctors’ use of apps at the various institutions. The aggregate pattern of individual behavior at the hospital level reflects its general pattern in different places. This study is also explorative and focuses on thoughts and opinions of doctors with regard to their choices of mobile apps. Thematic analysis was used to explore their perceptions of specific mobile apps, their patients and the hospitals with which they are affiliated. Then, we used the subjects’ responses for thematic analysis and made relevant comparisons across two cities.

### 2.2 Setting

We select two cities, Hangzhou (HZ) and Yancheng (YC), that represent two different stages of transforming society for the cross-region comparison. The former, the capital city of Zhejiang Province, is famous for hosting giant IT companies, such as Alibaba and Alipay. The growing economy there has attracted many people from across the country. YC, located in the northern part of Jiangsu Province, is a smaller city than HZ in terms of local population.

We chose to compare economic and demographic aspects of the two cities. First, both cities are in the Yangtze River Delta region, a comparatively rich area. The economic gap between the two cities is not large, as poor cities could hardly afford the internet infrastructure to support local doctors’ utilization of mobile apps. According to local data in 2021 from the local statistics bureau, the GDP *per capita* was 149,857 yuan in HZ, 98,593 yuan in YC, with 80,976 yuan for the national level (21, 22). Second, the local demographic character of the two cities is different. Many young people have moved away from YC to bigger cities, meaning that the older generation, especially those in the downtown area, have lived there since they were born, and are more likely to have established the strong social connections typical of “acquaintance society.” To be sure, there are recent arrivals in YC, however, the proportion of these people is much lower than the figure in HZ. The booming economy and better living conditions of the metropolitan HZ have attracted many newcomers, forming “stranger society.” According to data from the local statistics bureau, the resident population in HZ increased by 14.9% from 2015 to 2021 mainly due to an influx of young people, while the population of YC decreased by 7.7% in the same period because of migration to other big cities (21, 22).

### 2.3 Data collection and analysis

We chose tertiary hospitals for our institution-level comparison. In their study, Li and colleagues found that doctors in these hospitals are more likely to use online medical platform apps (23). The doctors in tertiary hospitals merit more research. We focused on three top tertiary (ranked as the national top 100 hospitals in China) and average (all the other tertiary hospitals) in HZ for the study. It must be noted that YC only has three average tertiary hospitals. Considering the medical professional advantages that the top tertiary hospitals enjoy, we listed this group separately. Thus, our study included three groups of tertiary hospitals: top facilities in HZ as well as average ones in HZ and YC.

Doctors are all obligated to attend the official medical platform apps built by their employers and doctors’ choices are the same, making the comparison unnecessary. We compared doctors’ adoption patterns of the most popular commercial medical platform in China, Haodf. The difference was measured by the average ratio of those using the app among all doctors and the average service counts per doctor at three groups of tertiary hospitals. The data were collected from Haodf website.^1^ Using PyCharm (Community 2022.3 version), we compiled a complete list of doctors who are using this app, the service counts of online medical consulting, and the hospitals with which they are affiliated. We then separated the doctors into three groups based on their hospitals. The percentage was calculated by dividing the number of doctors using the app in a particular group of tertiary hospitals by the total number of doctors in the group. Similarly, the service count per doctor was calculated by dividing the total number of online consulting sessions by the total number of doctors in the group.

This study also employed thematic content analysis of qualitative data from semi-structured interviews with selected doctors in HZ and YC. Inductive content analyses are used widely to research doctor-patient communication (17). We chose 10 doctors from each city under study. In HZ, we selected five from top tertiary hospitals and five from average ones. In YC, we interviewed 10 doctors from average tertiary hospitals.

All interviews were conducted from June 2022 to May 2023. Our semi-structured interviews were performed on the questions listed in Table 1. Each interview took from half an hour to an hour. With the interviewees’ permission, we recorded and transcribed all responses. Two researchers independently searched for topics from each interview, discussed their findings and reached a consensus on possible common themes. After analyzing 20 interviews, we found no new emerging themes.

**Table 1:** Semi-structured interview questions.

Were you born in this city?
What are the demographic characteristics of your patients?
Have you contacted your patients via WeChat? Are you willing to share your WeChat account with your patients?
Why do your patients choose you as their medical service provider?
Does the hospital with which you are affiliated discourage or support doctors’ use of medical platform apps?
Does the hospital with which you are affiliated have its own medical platform app?
With all the options available, how do you choose a particular medical platform app?
Do you feel pressured to attract more potential patients?

## 3 Results

### 3.1 Descriptive statistics

Table 2 lists the result of analyzing the data collected from the Haodf website. The average ratio of those using the app and the average service counts per doctor were the highest for those in the top tertiary hospital in HZ and the lowest for those in the average tertiary hospitals in YC. The two measures of average tertiary hospitals in HZ were both in the middle. The data, which covered all tertiary hospitals in the two cities, indicate that doctors in HZ use Haodf app more frequently at the time when data was collected. The service counts and the number of doctors using the app might keep changing in the future, however, these results are still useful for the recent years.

**Table 2:** The differences in doctors’ use of Haodf in three groups of tertiary hospitals.

Different groups of tertiary hospital	Average ratio of the doctors using the app^1^	Average service counts per doctor^2^
Top tertiary hospitals in HZ	49.97%	261
Average tertiary hospitals in HZ	41.00%	182
Average tertiary hospitals in YC	32.03%	39

^1^The data was collected on August 24, 2023, from the Haodf website. ^2^The data was collected on September 8, 2023, from the Haodf website.

### 3.2 Thematic analysis of the semi-structured interviews

Table 3 summarizes the basic information of these interviewees. Most interviewees are males, and their ages are in the 40s. They have different professional titles, mostly associate chief physicians, and are affiliated with different departments. We extracted three main themes from the interviewees’ responses. The first concerns physicians’ perceptions of the patients who live in the selected cities with certain characteristics. The second focuses on which mobile apps they choose and why. The third theme involves their observations on the role of hospitals in promoting mobile apps.

**Table 3:** Characteristics of participants (*N* = 20).

Participant characteristic	Values
Gender, n (%)	
Male	18 (90)
Female	2 (10)
Age, n (%)	
30–40 years old	4 (20)
40–50 years old	15 (75)
50–60 years old	1 (5)
Professional title, n (%)	
Chief physician	6 (30)
Associate chief physician	10 (50)
Attending doctor	4 (20)
Type of affiliated hospital, n (%)	
Top public tertiary hospital in HZ	5 (25)
Average public tertiary hospital in HZ	5 (25)
Average public tertiary hospital in YC	10 (50)
Affiliated department	
Surgery	7 (35)
Internal medicine	4 (20)
Traditional Chinese medicine	3 (15)
Ophthalmology	2 (10)
Dentistry	1 (5)
Neonatology	1 (5)
Cardiology	1 (5)
Dermatology	1 (5)

^1^The data was collected from June, 2022 to May, 2023.

#### 3.2.1 The interviewees’ perceptions of patients

In YC, eight of the 10 interviewees were born there and married local residents. They received their medical education in other cities and returned home to establish their practices. One interviewee stated that she and her husband, who were born and raised in YC, both come from big families, which provides even more connections (Interviewee ID: YC3). Many downtown residents turn to acquaintances when seeking medical services. For complicated disease treatments such as surgeries, turning to social connections is a natural choice for local residents. According to one interviewee, the ratio of surgery patients introduced by the acquaintance among all of his surgery patients is between 80 and 90% (Interviewee ID: YC4).

Due these strong social connections, the relationship between patients and doctors goes beyond providing medical services in YC. In other words, patients could also be doctors’ friends or friends’ friends. Whether there are extra connections between patients and doctors is one important criterion for doctors to classify patients. In one interviewee’s eyes, there are three kinds of patients: acquaintances, the strangers introduced by the acquaintance and the strangers with no referral from the acquaintance (Interviewee ID: YC7). However, these strong connections have been challenged by local demographic changes. First, an increasing number of doctors working in YC were not born there, as exemplified by the two interviewees from other cities (Interviewee ID: YC1 & ID: YC2). Second, more outsiders from northern or western provinces have come to live in YC. Third, many patients in rural YC might seek doctors in the city, bypassing local primary health care agencies and county hospitals. All interviewees agreed that the proportion of strange patients (without connections to the acquaintances) has been increasing.

Strong social connections do not guarantee good communication between doctors and patients., The doctor-patient relationship may still be strained in YC. As indicated by Table 4, doctors may have challenges with some patients, including the sacrifice of personal off-duty time to provide consulting services to patients, the loss of patients to hospitals in big cities, and the possible disputes with patients.

**Table 4:** Interviewees’ challenges with patients s in YC.

Different challenges	Select interviewees’ responses
Off duty consulting	*“Many of my older patients call at 9. pm or even later for medical advice.”* (ID: YC3)
Pursue of the over-qualified hospitals	*“Many local residents seek medical services in big cities in Southern Jiangsu or Shanghai for routine or unnecessary chest surgeries.”* (ID: YC4) *“Some local patients believe that medical services are better in Shanghai. They have vision problems that are easily correctable, and yet they still go to Shanghai.”* (ID: YC 10)
Possible disputes with patients	*“Some patients are terrible. They not only argue with you, they hit you.”* (ID: YC2)

In HZ, none of the 10 interviewees were born in HZ, and their acquaintances include their colleagues, family members, friends, and long-term patients. They have far fewer family members nearby than their counterparts in YC, and their patients are more diversified in terms of their geographical locations. For average tertiary hospitals, patients are mostly from HZ. For top tertiary hospitals, many patients are from other cities in Zhejiang province or other provinces.

Even though the composition of patients in HZ differs from YC, the doctors might still have similar challenges as their counterparts in YC. Table 5 lists three challenges for doctors when communicating with patients. The types of challenges might be similar between the two cities, but the doctors’ personal reactions toward the patients might be different. Doctors in HZ are less likely to share their cell phone numbers with unconnected patients, and none of those in top hospitals are willing to do so. The interviewees must distance themselves from their patients to protect their personal lives. They have a stronger sense of keeping the boundary between professional work and personal life than those in YC, as indicated by one doctor’s response (Interviewee ID: HZ10 in Table 5).

**Table 5:** Interviewees’ challenges with patients in HZ.

Different challenges	Select interviewees’ responses
Off duty consulting	*“I give my personal phone number to my patients. Most have common courtesy, and only a few are very bothersome. If they call me later at night, I do not respond and block their phone numbers.”* (ID: HZ8) *“I do not want to be bothered after work. I do not have the obligation to do that.”*(ID HZ10)
Pursue of the over-qualified hospitals	*“People with no medical training are not good at choosing the appropriate doctors to treat their disease. Among my acquaintances, my classmates in middle school who have limited medical knowledge frequently seek medical services in Shanghai. My colleagues in this hospital would refer their acquaintances to me.”* (ID: HZ2)
Possible disputes with patients	*“Some patients are unreasonable, and we must be very careful when communicating with them. Based on my experience, those who are well-educated, government employees or teacher have better medical compliance, and it is much easier for me to communicate with them.”* (ID: HZ6)

#### 3.2.2 Interviewees’ use of mobile apps to communicate with patients

Participants have used different kinds of mobile apps to communicate with their patients. Table 6 summarizes their choices. According to the table, WeChat is the most frequent app used to communicate with patients. WeChat is a popular social networking app in China, and users can share messages, pictures, and videos with others. According to Table 6, those in top tertiary hospitals are more likely to use the medical platform apps (including the official medical platform app of their affiliated hospitals and the commercial medical platform app such as Haodf). Their hospitals could build their internet hospital in the form of a medical platform app or WeChat’s mini-program. Internet hospital is a term referring to online programs combining offline and online access to medical resources, and patients can register, have prescriptions, review their medical records, or have online communications with their doctors (24). These mini-programs are similar to these hospitals’ official medical platform apps regarding their functions. All of them have taken part in their hospitals’ official platform apps, though the chances to use them vary. Twelve of the 20 participants have chosen the commercial medical platform app of Haodf.

**Table 6:** Interviewees’ choice of mobile apps for communicating with patients.

Different kinds of tertiary hospital	One-to-one WeChat with strangers	One-to-one WeChat with those patients introduced by acquaintances	Using the official medical platform app of their affiliated hospitals	Using the commercial medical platform apps
HZ Top tertiary hospitals	None	5 out of 5 interviewees	Frequently use.	4 out of 5 interviewees.
HZ Average tertiary hospital	2 out of 5 interviewees	5 out of 5 interviewees	Rarely use.	3 out of 5 interviewees.
YC Average tertiary hospital	5 out of 10 interviewees	9 out of 10 interviewees	Rarely use.	5 out of 10 interviewees

In YC, the interviewees are more likely to use WeChat to communicate with unconnected patients than their counterparts in HZ, with only one exception (ID YC2), who moved from another province to YC. He declined to share his WeChat account with anyone, even those introduced by his friends for two reasons:

“*the first reason is that the current patient-doctor relationship is too bad. I had fights with my patients. I do not want to incur any trouble in these unofficial communications; secondly, I have already been very exhausted from my daytime job. I work more than 60 hours a week. I have one night shift every five days. The income is insufficient to force me to sacrifice my off-duty time.*”

Among the interviewees in YC, those in specialties such as dermatology, internal medicine, and ophthalmology, are more likely to use commercial medical platform apps. For example, one ophthalmologist had more than 1,000 communications with patients (ID: YC10). Surgeons are unwilling to adopt commercial medical platform apps out of the concern for being at risk. Patients, particularly those in need of surgeries, have complicated medical conditions, and the online conversations could not provide a comprehensive description. One interviewee is from a surgery department and his online consultation in Haodf is less than 10 (ID: YC5).

*“I treat many different types of eye disease. I have out-patients every workday. Our surgeries are very simple and I do not need cooperation from my colleagues.”* (ID: YC10)

“*I use Haodf, but very rarely out of the fear of the potential risk resulting from the online communication.*” (ID: YC5)

In YC, five out of 10 interviewees do not use commercial medical platform apps because of a variety reasons, some of which are listed in Table 7. Among those using Haodf, two physicians (ID: YC9 & YC10) have more than 1000 rounds of online medical consultation with the online patients. According to their response in Table 7, they both choose the app to conveniently communicate with patients. For the official medical platform apps or mini programs in WeChat from their employers, they are obligated to use. However, all of them seldom have the chance to use them because most of the local patients are unaware of these communication tools. The three hospitals started to build their own internet hospitals (either mini programs in WeChat or mobile apps) after the outbreak of COVID-19.

**Table 7:** Reasons interviewees in YC choose to use or reject commercial medical platform apps.

To use/reject	Select interviewees’ responses
Reject	*I have too much work, and adopting new mobile apps requires extra time and energy which I could hardly afford. Besides, online consults are much less comprehensive and effective than offline services.* (ID: YC3)
Reject	*YC is a small city. Our patients are mostly local residents, and it is very convenient for them to visit our hospitals. They prefer to face-to-face communication, which is more convenient. Besides, they are unwilling to pay out-of-pocket fees for the online consulting service.* (ID: YC6)
Reject	*I have doubts about the online information. I just started using Ali Pay, and the online medical platform apps are unreliable.* (ID: YC5)
Reject	*Local residents have one or two acquaintances who work in hospitals. They could call or use WeChat to communicate with medical professionals if they have any questions. They do not have to pay for the online services provided through commercial medical platform apps.* (ID: YC1)
Use	*I started to use commercial medical platform apps when I was in medical school to pay for my tuition. During the 3 years of COVID-19, I had fewer face-to-face consults and turned to online apps to make enough money for my mortgage.* (ID: YC2)
Use	*I have had an increase in patients from rural areas in the last 10 years. I do not want to share my personal phone number and WeChat account with them. Haodf allows me to communicate with them without sacrificing my privacy. I also work with different kinds of patients with the help of this app.* (ID: YC10)
Use	*I learned about Haodf from my graduate advisor and it is very easy to use for us dermatologists.* (ID: YC9)

In HZ, all interviewees have used their personal WeChat to communicate with patients, though some are more selective for adding unconnected patients to their personal accounts. According to Table 6, those from top hospitals are less willing to share their account information than those from average tertiary hospitals. Like their counterparts in YC, physicians must participate in their hospitals’ official online service (either mini programs in WeChat or mobile apps). Seven out of 10 interviewees in HZ have been using commercial medical platform apps, of which four are from top hospitals. Table 8 lists some of their responses to the reasons for using or rejecting these commercial apps. According to Table 8, medical risk and the limited energy are still the reasons for some participants to reject the apps, while some would turn to the apps for providing the convenience to patients, or for attracting new patients. According to the interviewees’ responses, promoting their individual or their department reputation has been popular among those using commercial apps (Interview ID 3, 8, and 9 in Table 8). The comparison of responses in two cities suggests that reputation marketing is more important in HZ. One interviewee indicated,

**Table 8:** Reasons interviewees in HZ choose to use or reject commercial medical platform apps.

To use or not to use	Select interviewees’ responses
Reject	*The medical platform cannot protect us when we have disputes with some unreasonable patients.* (ID: HZ6)
Reject	*First, I am too busy with offline services. Second, online communication is risky. In addition to caring for my offline patients, I also provide advice to patients recommended by my acquaintances. My time is limited.* (ID: HZ7)
Reject	*There are too few online patients. I would like to build my offline reputation first and consider using it in the future.* (ID: HZ4)
Use	*We have many patients from other cities in Zhejiang or from other provinces. They do not have to come to our hospitals for follow-up consultations. We use apps for their convenience so that they can contact us with regard to their medical exam results or the latest medical conditions.* (ID: HZ2)
Use	*As the department director, I encourage young doctors to use the apps to get more income and attract more patients from online consulting to our offline services.* (ID: HZ9)
Use	*As the founder and director of our new department, we need to compete with other similar departments in other top hospitals. I use the apps to promote our department’s reputation. I also have a Douyin account^1^to which I have uploaded more than 100 hundred short videos. I now have more than one million subscribers.* (ID: HZ3)
Use	*I use the apps to educate my patients about the many uses of acupuncture. People are not very familiar with it. I need to let patients know what kind of disease I could treat.* (ID: HZ8)

^1^Douyin is a Chinese version of TikTok, a short video-sharing app.

*“In big cities such as HZ, reputation marketing is very important for us. Doctors in small cities might not be so concerned with reputation marketing.* (ID: HZ8)”

#### 3.2.3 Perception of hospitals’ use of mobile apps

According to the participants, the three tertiary hospitals in YC, the top and average tertiary hospitals in HZ all actively promote mobile apps to attract patients. All of them have built their internet hospitals via the mini program in WeChat. One has even created an independent app similar to Haodf. None of these tertiary hospitals have prevented their employees from using commercial medical platform apps.

Before the pandemic, only the top hospitals in HZ were actively in establishing their internet health programs. However, after 3 years of strict COVID-19 control policies that included extra restrictions on hospital access, average tertiary hospitals have turned to online services in addition to the offline services, which have decreased dramatically in both HZ and YC. As one department head (ID: HZ9) responded in Table 8, the department and the hospital hope these online consultations will help divert patients from online communication to offline services.

In addition, medical platform apps have become popular due to fierce competition among tertiary hospitals in both cities. Table 9 lists some responses from these interviewees. The pressure from fierce competition has been passed down to the heads of each department. One of the interviewees (a department head) responded,

**Table 9:** Interviewees’ perception of pressure to attract more patients.

Potential competitors	Position in hospital	Select interviewees’ responses
Competition among local hospitals in YC	No administrative position	*“Previously insurance plans restricted patients’ choice of local tertiary hospital. Current insurance plans allow local residents to choose a hospital. The three biggest hospitals in YC are competing fiercely.”* (ID: YC3)
Competition between YC and Shanghai	Department head	*Doctors in Shanghai have many more patients than they can handle.* (ID: YC10)
Competition between top hospitals and average tertiary hospitals	No administrative position	*The top hospitals have added branches and have attracted many patients who had previously sought services in our hospital, and the pressure for us is overwhelming.* (ID: HZ7)
Competition among top hospitals in neighboring cities.	No administrative position	*Our hospital is the best hospital in HZ, and yet we still have the pressure to attract more patients. There is a limited number of Gamma Knife radio surgery centers in Europe. However, we have several centers in the Yangtze River Delta covering HZ, Nanjing, and Shanghai. The competition among the top hospitals is even more intense* (1). (ID: HZ1)

^1^These big cities, HZ, Nanjing, and Shanghai, are close to each other, less than 2 h ride.

“*My biggest pressure is to attract and keep patients. Nowadays, the pressure is much more intense than before. However, the doctors in our department are not enthusiastic about using medical platform apps or short video apps to attract patients.*” (ID: YC10)

In order to attract as many patients as possible, these hospitals have also created WeChat public accounts to reach out to the public and promote their medical services. Not only is each department required to have its own public WeChat account to regularly circulate health education articles, all department heads in the two cities are required to use their personal WeChat accounts to circulate these articles (ID: HZ3; HZ9; YC9; YC10).

Table 9 lists different kinds of competition that these doctors experienced. According to interviewees’ responses in this table, while hospitals in both cities are pressured to attract more patients, this is particularly true in YC, as more local patients are more likely to choose well-known institutions in Southern Jiangsu province and Shanghai. This pressure is also evident in HZ, even for the top ones. According to Table 9, the competition has also driven top hospitals to import more advanced medical equipment, such as Gamma Knife (a costly radiation therapy device), as one interviewee responded (ID: HZ1). The top hospitals have also been more active than other hospitals in terms of their involvement in using the medical platform apps. One out of the three top hospitals built its own mobile apps and the other two built their internet hospitals in WeChat mini program even before the outbreak of COVID-19. As shown in Table 2, these doctors have the highest percentage of commercial apps use and service counts of online medical consultations.

## 4 Discussion

### 4.1 Findings

Based on the descriptive statistics, a comparison based on different tertiary hospitals suggests doctors in HZ are more likely to use commercial medical platform apps than their counterparts in YC. Among the former, those from top hospitals use medical platform apps more frequently than those in average ones. Previous studies concur that doctors from tertiary hospitals are more likely to utilize mHealth apps than those from non-tertiary ones (23), and yet they seldom indicate the potential differences among tertiary hospitals.

Thematic analyses of responses from selected interviewees in the two cities indicates that the stronger social connections between doctors and patients frequently found in YC has led to more complicated relationships. According to one previous study, only 68.6% of general practitioners would add an unconnected patient to their personal account (25). In our study, only two out of 10 in HZ and five out of 10 in YC were willing to do so. This lower figure might be from the different types of doctors. Our interviewees are all specialists in tertiary hospitals who are less likely to share their accounts than general practitioners. However, 19 out of 20 interviewees are willing to add a patient to their account if they are recommended by an acquaintance. Surprisingly, this common phenomenon has not yet been addressed in previous research. Our study fills this gap by focusing on the doctors’ choices between social networking apps and the medical platform apps.

By comparing internal differences in interviewees’ responses in each city, we found that those using medical platform apps are more likely to treat chronic diseases and perform surgeries more independently (i.e., dermatology or ophthalmology department) than those in need of more cooperation (i.e., chest surgery department) among colleagues in YC; doctors from top tertiary hospitals are more likely to use medical platform apps than those from average ones in HZ.

We found the doctors in HZ and YC have adopted medical platform apps for greater conveniences and ease of establishing an online reputation. Some doctors are unwilling to try this new technology because they are too busy with offline patients and fear potential risks (Tables 7, 8). These facilitating and preventive reasons have been documented in existing studies (26, 27). In our study, we found that the patterns of social interactions might also affect the interviewees’ choice, which is rarely addressed in the extant studies (2).

Heavy competition for patients has inspired physicians to utilize these medical platform apps. On one side, these hospitals have all established their medical platform apps, either in the form of an independent mobile app or in the form of a mini program in WeChat. On the other side, they encourage their employees to participate in commercial medical platform apps. The top hospitals have made more progress than other average tertiary hospitals. This finding is consistent with other studies, indicating that top hospitals are often front runners in the adoption (24, 28).

### 4.2 Society’s impact on doctors’ app adoption

Our findings indicate that society plays a significant role in understanding the doctors’ adoption and use of the apps. In similar research, Zhang and colleagues allude to an immediate social environment, which was defined by a series of questions related to peers’ choice of the apps, and macro social factors, exemplified by users’ type of medical insurance (29). In Wu’s study of the patients’ use of medical online apps, the social factor is defined as support from a peer group or the possibility of using the app to interact with other users (30). In both studies, the social factors are viewed exclusively from the demand side; however, they could be the property of the whole society in general. Compared to existing studies on social influence, our study focuses on the wholistic nature of society, which affects all members including doctors and patients.

In this part, based on the above findings, we elaborate on the reasoning mechanism behind individuals’ choices and motivations. The social environment is a varying variable. Traditional and modern societies have very different patterns of social interactions. However, they are not mutually exclusive. For example, in HZ, social connections still have subtle impact on the doctor-patient relationship. As two cities experiencing their transforming process from traditional society to modern society, HZ is closer to modern society than YC. Below, we discuss the basic logic of the doctors’ adoption behavior in the two cities.

In addition to providing further understanding of doctor’s use of apps in transforming society, the macro and wholistic view could help identify the change of other elements in transforming society, such as medical service.

#### 4.2.1 The logic of using social connections in traditional society

There are many specific reasons behind the doctors’ adoption of a particular mobile app. Behind these reasons, maximizing the utility of their medical knowledge and service is the key motivation to understand doctors’ behavior. Hsiao claims that medical professionals’ self-interest is the key to understanding the healthcare reform in China (31). This basic assumption could also be used to understand doctors’ communication with patients. There are two kinds of returns deriving from this basic motivation: quick returns such as financial returns and long-term returns such as exchanged benefits from long-term cooperation or more recognition by others from accumulated reputation.

According to the participants, doctors in YC are willing to use their WeChat or their personal phones to respond to patients, because social connections are stronger in a small city and patients are more likely to be acquaintances or friends of acquaintances. Although doctors’ greater investment of energy and time does not result in direct returns, such as fees from the online medical platform apps, strong social connections with relatives, friends, neighbors, colleagues, and other acquaintances strengthen long-term cooperation and the obligation to return favors. For example, these doctors might turn to their relatives or friends for tutoring for their children or household repairs. Social connections are established and strengthened by doctors exchanging their particular professional knowledge with those who remain in the social circle long term, a scenario which forms acquaintance society (32).

In HZ, close social ties have been broken due to an increase of strangers migrating to this city. There are many requests for certain social needs in a big city, which makes the utilization of social connections more difficult with strong surveillance from the public (strangers as a whole). The utilization of social connections is against the rule of fairness for the general public since every patient needs to be treated equally. These interviewees are more likely to choose fees from the medical platform apps because they have not had the opportunity to form strong social connections. None of the interviewees were native to this metropolitan city.

However, medical services in HZ still use social connections, which do influence doctor-patient relationship. All participants have shared their WeChat accounts with patients who were introduced by those in the social circle, though some of them are more selective. Social connections are essential in the medical field as colleagues (mostly specialists as well) exchange information and medical advice that help them build strong associations with other doctors in other departments, thus increasing the utility of their medical knowledge of both parties. For example, one doctor in the ophthalmology department might seek help for his/her relatives from his/her colleague in the dental department and serve as the trustful middleman for two parties. By exchanging their medical suggestions, they could expand the circle of their friends by attending to more types of medical requests.

In order to make the most of their medical knowledge and services, doctors must establish a reputation of being knowledgeable and trustworthy for long-term success. Exchanging different professional advantages with one’s friends in other professional areas (i.e., teachers) or exchanging treatment information with doctors in other departments is presumed with one basic condition-- this doctor has already won recognition and the trust of his or her friends or colleagues in previous rounds of interactions.

**Figure 1:**
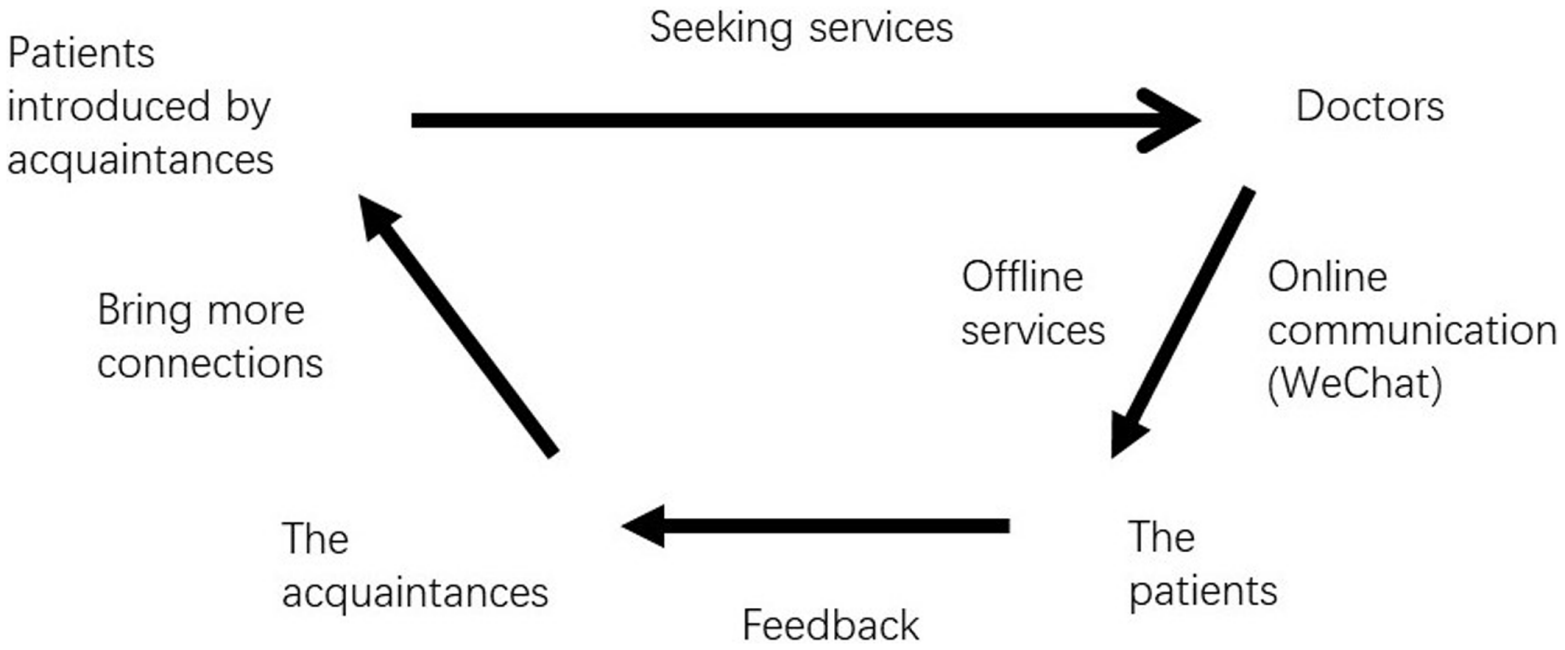
The model of using social connections to seek medical services.

Reputation building is important for medical professionals because patients and their families care about the quality of medical services. In traditional society, the essential task of reputation building takes a long time. Figure 1 presents a model demonstrating reputation building via social connections, in which an acquaintance connects doctors and patients. Positive evaluation of services could attract new patients. As hinted from this figure, doctors build their reputations for performing multiple services and his/her specialty can only be recognized by those close to him/her, such as colleagues or his/her friends. It is unlikely for outsiders to have access this vital information. Thus, reputation building in traditional society is not as prominent as its counterpart in modern society.

#### 4.2.2 The logic of using medical platform apps in modern society

Unlike the counterpart in traditional society, doctors may not know many of their patients in modern society. Building long-term trust between them is not easy in China. In previous studies, scholars have found that with trustworthy middlemen, it is easier for doctors and patients to build interpersonal trust (32). The way that doctors maximize the utility of their professional knowledge in modern society is different from how they do so in traditional one. To increase the utility of their medical services and knowledge, they need to cash their medical knowledge and services more quickly and to build their reputation at a larger scale. They could turn to medical platform apps to reach these goals instead of turning to social connections. Scholars have found that reputation building strongly incentivizes doctors to use medical platform apps (33). The interviewees’ responses in our study provided further support for this suggestion.

**Figure 2:**
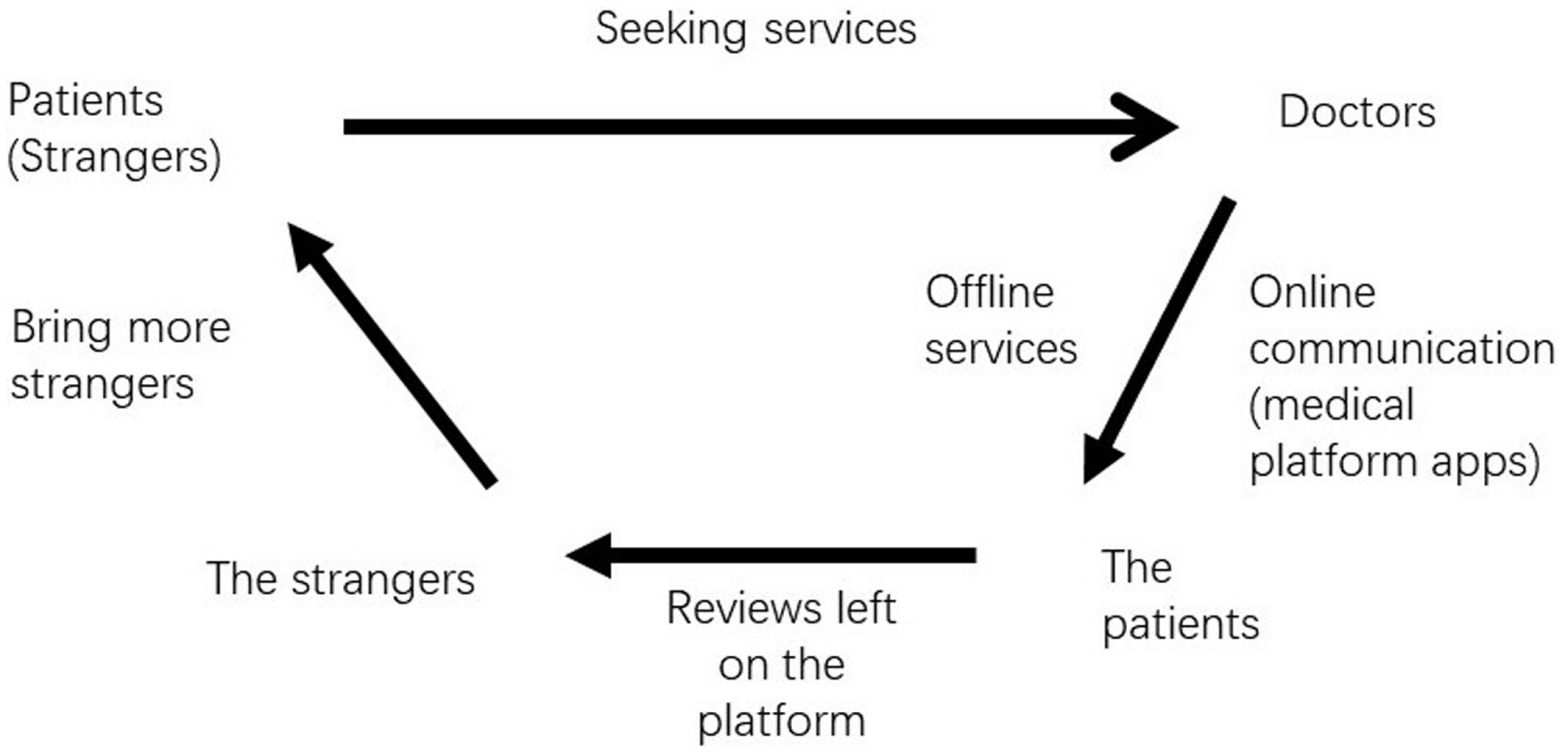
The model of using medical platform apps to seek medical services.

Figure 2 illustrates the process of building a reputation through these platforms. Unlike social connections, this model suggests that patients’ evaluations could be accessible in medical platform apps to those looking for doctors, which could attract more patients with similar medical needs. The number of these potential patients is larger than the number of patients introduced by acquaintances. In HZ, doctors work with more patients outside the social circle and adoption of medical platform apps help them build a more professional relationship with patients. The percentage of these unconnected patients is higher in top hospitals than those in average ones. Thus, physicians working in top hospitals are more likely to use the apps, a finding which is supported by descriptive statistics and thematic analysis of semi-structured interviews.

“Being too busy to learn to use medical platform apps” is a common reason that interviewees, particularly surgeons in both YC and HZ average tertiary hospitals, choose not to use them. They are occupied with in-patient and out-patient care and do not have as much time to spend with patients as do dermatologists or ophthalmologist. However, the busiest surgeons in top hospitals still choose to work with the apps to provide more services to their patients. In previous studies, scholars have also found no statistical relationship between workload and doctors’ adoption of online medical platform apps (33). Our study provides some evidence for this conclusion.

Even in YC, some doctors actively use commercial medical platform apps. One interviewee (ID: YC2), a newcomer, does not want to build strong social connections in this new city. With Haodf, he can make more money to pay his mortgage and build his long-term reputation in online apps among strangers. An ophthalmologist and dermatologist (ID: YC9 and ID: YC10) mentioned that they have used the commercial apps for over 1,000 online consultations, surpassing all interviewees in HZ. They could conduct these surgeries without their colleagues’ help, which means they could provide services to more patients than those doctors in normal surgery departments. According to these two interviewees’ responses, they have more patients from rural areas, who chose these two interviewees based on the other patients’ evaluations on the app.

Medical platform apps are not only an important tool for reputation building for individual doctors but also for marketing and establishing a good reputation for the institutions for which they work. As shown in Tables 2 and 6, better hospitals attach greater importance to online services. These hospitals have developed more online health programs than average tertiary hospitals, which is consistent with other scholars’ findings (34). In addition, app use is highest among doctors from top hospitals. The offline advantages enjoyed by these top hospitals have been expanded to their online services.

As previously stated, these apps are critical to building reputations for the hospital as well as for individual department. Four department heads in HZ and YC are active app users (ID: HZ 3, HZ 9, YC 9, and YC 10) and strongly encourage doctors in their departments to do the same. Moreover, they have created a WeChat public account to market the services of each department.

### 4.3 Two transformations

Under the context of transforming society, China has embraced many technological conveniences, including medical platform apps. Many young people are moving into HZ, which is experiencing an economic boom. They have broken down the traditional comparatively closed society and thus the pattern of social interaction. Thus, the doctor-patient relationship is more professional in this city because it is less likely to be complicated by other social connections, which are typical in traditional societies. Doctors in this city are more likely to use medical platform apps to communicate with their patients and to build their personal reputations without the need to sacrifice personal privacy.

In YC, the number of newcomers is too small to change the pattern of old-style social interactions. Locals typically turn to social connections for medical services. Doctors in YC are more likely to use personal phones or WeChat accounts to communicate with patients. However, according to some interviewees, professional relationships are becoming more common, largely due to medical platform apps through which doctors build a professional reputation. As demographic conditions keep changing in YC, more and more doctors and patients will accept these technological innovations (ID: YC9 & ID: YC10).

Medical service is also experiencing a transformation from a closed model to a more open one because patients have greater mobility and can choose doctors further away. Thus, doctors in both cities now face more pressure to attract new patients. This pattern has also appeared in other studies by scholars who found that internet searching increases the patients’ tendency to go to higher-level medical institutions because they are inundated by online information and are nervous about their possible medical conditions (35).

This study presents a different explanation. In YC, comparatively traditional society, the interviewees reported the loss of patients to other hospitals in the same city or those in other big cities, as suggested in the discussion of Table 9. Previously, the patients’ choices of medical service were limited because they had been attached to their acquaintances, who would mostly help them find the local service providers. According to the literature of the transforming society (8, 9), individuals have become increasingly independent on social interactions. Patients have become more independent when seeking medical services, which has led to a more open model of the medical service market. However, the transformation process is long and relying on social connections still exists (12). Even in Hangzhou, interviewees admitted their adoption of WeChat to provide informal suggestions to patients introduced by the intermediaries, as indicated by Table 6. The findings from both cities demonstrate that the utilization of social connection or not has become a key variable to understand individual’s medical service behavior in China.

According to the literature on the transforming society, traditional society and modern society have respective characteristics, particularly in the pattern of social interaction (8, 36). Individuals in traditional society are more likely to utilize personal connections than those in modern society, and the progress into a more modernized society means people become less dependent on intermediaries (36). The findings of this study provide empirical support for this trend. A transforming society could attract new patients outside the social circle to a city and thus affect how doctors communicate with their patients in general. In traditional society, strong bonds are formed between doctors and patients as a result of local social connections. In modern society, the fluidity of patients has increased because these bounds weakened to some extent. These patients may now independently choose an appropriate doctor. Other considerations being equal, they are more likely to choose better doctors in better hospitals, though they might not know these doctors directly or indirectly. Thus, the reputation of being a good doctor in an open platform is important for doctors, as suggested in some interviewees’ responses in Tables 7, 8. The weakening bonds between acquainted people and the increasing emphasis on the open reputation among strangers in a transforming society can also be utilized to explain consumers’ behavior in other online platforms such as Taobao, the biggest online shopping platform in China (37, 38).

Other elements might also add to these changing patterns. First, the Yangtze River Delta region, encompassing Jiangsu, Zhejiang, Shanghai and Anhui, is one of the most economically developed areas in China. Wealthy residents have more financial resources to purchase quality medical services. Second, the reform of insurance plans allows patients to choose their hospitals. For example, patients in the Yangtze River Delta region may seek hospitals in other cities or provinces in this area. Shanghai, which possesses the most advanced medical service institutions, has attracted many patients from both YC and HZ. Third, transportation in China has made impressive progress. With the help of high-speed railways, patients can easily travel to hospitals in metropolitan areas. Finally, medical platform apps provide a service guide to help patients seek distant medical services.

In modern society, people do not have to turn to acquaintances to find doctors. For patients, medical platform apps serve as alternative guide; for doctors they are a tool for building their reputation to attract more patients. Mesko and colleague proposed the possibility of “empowered” physicians in the digital health era (39), and our findings demonstrate this empowerment. To a certain extent, online medical apps have taken the role of social bonds.

## 5 Theoretical contributions and policy implications

Using a mixed-methods design, we found that doctors in YC are less likely to adopt medical platform apps than their counterparts in HZ and turn to social networking apps to maintain their connections with their acquaintances, reflecting the continuing influence of traditional society. However, traditional social interaction and the patient loyalty are facing challenges. Empowered patients are able to vote with their feet, leading to changes in the demographic composition of patients. In transforming society, with more people migrating to new places, social connections begin to collapse, and medical services are becoming market-oriented or patient-centered. The medical platform apps serve as a catalyst in this process.

This study contributes to the extant literature in two ways. First, our research compares doctors’ adoption of mobile apps in two cities. Unlike previous studies focusing on individual attributes (40), our study places the individuals in a particular social context. Under the inspiration of the transforming society perspective (8, 12), our study assumes that regions have different characteristics, which might affect the doctor-patient relationship in general and the doctors’ adoption of mobile apps to communicate with patients in particular. Our findings provide further empirical support for the transforming society views.

Second, our study contributes to the cross-regional research of users’ adoption behavior of mobile apps. Currently, although medical platform apps are becoming increasingly popular worldwide, cross-regional studies of adoption patterns are limited. Previously, scholars have explored the differences from the demand side, including comparing developing and developed countries or different regions in a particular country, such as China (41, 42). Hao and colleagues reviewed Chinese and American patients’ comments posted on two popular online medical platforms in each country, finding that the former commented more about medical treatment, while the latter focused more on recommendations. This may be due to the differences between the two countries’ health systems (41). Jiang and colleagues found that visits to medical platform are not distributed evenly among provinces in China (42). Our study added more discussion on the regional comparison literature.

In addition to cross-regional studies, some scholars have explored local differences in medical professionals’ use of apps (23, 43). Doctors using Haodf are mostly from provinces and cities in eastern China (23). Some scholars might attribute geographic difference in usage to the uneven distribution of regional medical resources, such as tertiary hospitals (43). These studies focus on the supply side or the demographic characteristics on the demand side. However, an exploration of the wholistic nature of a particular region that simultaneously affects the demand and provision aspects is missing in these studies. This study could fill in this gap.

The findings of this study have policy and practice implications. First, the findings suggest that doctors and patients in small cities are still less likely to use medical platform apps to communicate with patients. In these places, local hospitals and health care administrating agencies need to invest more financial and human resources to help doctors adopt these apps to provide patients with more convenience. Second, the findings suggest that doctors in both cities might use mobile apps to communicate with patients. Hospitals and relevant administrative agencies need to remind doctors that it is important to be professional when communicating with patients. Doctors need to protect patient’s privacy in online communication. Besides, online communications are not void of medical risks, and doctors need to be particularly careful when giving medical suggestions, especially for patients without previous medical exam results.

## 6 Limitations

This study is limited in three areas. First, the findings from the cities, doctors, and hospitals that were purposely selected for this study might not be applicable to other places in China. Second, we focused exclusively on Haodf, the most popular online medical platform in China, disregarding other available platforms. Third, the thematic analysis of semi-structured interviews is still from the interviewees’ personal narratives. It is not unlikely that, absent a reliability check, their narrative could be subject to distortion intentionally or unintentionally. However, the mixed-methods design is beneficial as a tool for macro-level analysis of the impact of social bonds and technology on the doctor-patient relationships, especially considering this impact has seldom been addressed in the current studies. Future research could extend our findings and explore more regional gaps in local medical professionals’ adoption of medical platform apps.

## 7 Conclusion

This mixed-methods study is a comparison of two cities to explore the reasons behind doctors use of mobile apps to communicate with patients. Doctors turn to social networking apps or online medical platforms or both. Other conditions being equal, doctors in traditional society are more likely to use social networking apps to communicate with patients, while those in modern society typically use online medical platform apps.

Our findings are not limited to China, as many developing countries are experiencing a transformation from traditional to modern society. Our study sheds light on the potential impact of society as a whole. Currently, the medical landscape is replete with technological advancements and innovations; therefore, it is much more important for “old school” doctors and patients to accept these technical innovations in transforming societies. It is important for researchers to explore the underlying traditions of the social environment and to understand the pattern of people’s social interaction, so as to help technology innovation such as digital health apps to take root in people’s minds.

## Data availability statement

The raw data supporting the conclusions of this article will be made available by the authors, without undue reservation.

## Ethics statement

Ethical approval was not required for the studies involving humans because this research does not involve participants who could be considered to be vulnerable. The studies were conducted in accordance with the local legislation and institutional requirements. The participants provided their written informed consent to participate in this study.

## Author contributions

DC: Writing – original draft, Writing – review & editing. ZS: Writing – review & editing, Conceptualization. ZG: Writing – review & editing, Conceptualization, Formal analysis, Funding acquisition.

## Footnotes

^1^https://www.haodf.com/
